# Isothiocyanates from *Brassica* Vegetables—Effects of Processing, Cooking, Mastication, and Digestion

**DOI:** 10.1002/mnfr.201701069

**Published:** 2018-07-12

**Authors:** Teresa Oliviero, Ruud Verkerk, Matthijs Dekker

**Affiliations:** ^1^ Food Quality and Design Group Department of Agrotechnology and Food Sciences Wageningen University Bornse Weilanden 9 6708 WG Wageningen The Netherlands

**Keywords:** digestion, glucosinolates, isothiocyanates, mastication, processing

## Abstract

The formation of health‐beneficial isothiocyanates (ITCs) from glucosinolates depends on a wide variety of plant‐intrinsic factors (e.g., concentration of glucosinolates, activity of myrosinase, and specifier proteins) and on a multitude of extrinsic postharvest factors such as the conditions used during industrial processing, domestic preparation, mastication, and digestion. All of these factors contribute to a large variability in the formation of ITCs (and other breakdown products), as well as their intake and absorption upon consumption of *Brassica* vegetables. This uncertainty in ITC intake and absorption is a barrier for the determination of an optimal *Brassica* vegetable consumption pattern. In this review, the intrinsic and extrinsic factors that affect the formation, intake, and absorption of ITCs are described according to the most recent findings. The focus of this review includes the hydrolysis reaction mechanisms, the elucidation of the primary factors that play a role in the hydrolysis reaction, the influence of processing and cooking conditions, the effect of chewing, and the roles of the gastric and upper intestinal phases, including the effect of the meal composition (e.g., the effect of other meal compounds present during digestion) on the potential formation of ITCs.

## 1 Introduction

**Figure 1:**
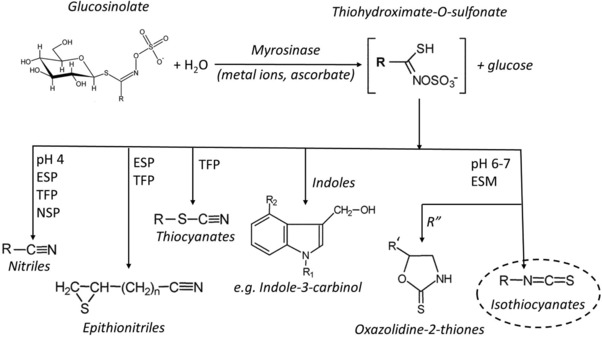
Chemical structure and breakdown pathways of glucosinolates. ESP, epithiospecifier protein; TFP, thiocyanate‐forming protein; ESM, epithiospecifier modifier protein; NSP, nitrile‐specifier proteins; R, variable side chain; R′, alkenyl side chain; R″, R′‐CH(OH)CH_2_‐ side chain.^1^, ^105^, ^106^

Glucosinolates (GLs) and their breakdown products (BDPs) have been extensively investigated for their beneficial effects on human health. GLs are relatively stable compounds that can be hydrolyzed by myrosinase (MYR), a β‐thioglucosidase present in GL‐containing plants and in the human gut microbiota, to form different BDPs. The type of BDP formed depends on the type of GL, the reaction conditions (e.g., pH), and the presence of certain specifier proteins (**Figure** 1).

Initially, research on glucosinolates has mainly focused on the toxic, anti‐nutritive, and goitrogenic properties of the BDPs.^1^ More recently, the focus has shifted to the study of BDP compounds that show beneficial health effects against various chronic diseases. The biological activities of the BDPs are varied and include modulation of xenobiotic metabolism and inflammation, regulation of apoptosis, cell cycle arrest, angiogenesis, metastasis, and regulation of epigenetic events.^1^ These potentially health‐promoting effects of *Brassica* vegetable consumption are supported by many epidemiological studies, as well as by in vitro and in vivo studies.^1^ Among the BDPs, the beneficial bioactivities are mainly ascribed to the isothiocyanates (ITCs).^2^, ^3^, ^4^, ^5^, ^6^, ^7^, ^8^, ^9^, ^10^, ^11^, ^12^, ^13^, ^14^, ^15^, ^16^, ^17^, ^18^, ^19^, ^20^

For this reason, researchers have worked on breeding *Brassica* varieties containing elevated levels of GLs leading to an assumed higher intake of BDPs.^21^, ^22^ However, a higher GL content does not guarantee an increased formation of the desired ITCs upon consumption of these vegetables. The formation of health‐beneficial ITCs from glucosinolates depends on a wide variety of plant‐intrinsic factors such as the concentration of glucosinolates and the activities of myrosinase and specifier proteins, and by a multitude of extrinsic postharvest factors such as the industrial processing conditions, domestic preparation, mastication, and digestion. All of these factors contribute to a large variability in the formation of ITCs and other breakdown products, their intake and absorption upon consumption of *Brassica* vegetables. This uncertainty in ITC intake and absorption is a barrier for the determination of an optimal *Brassica* vegetable consumption pattern.

In this review, the intrinsic and extrinsic factors that affect the formation, intake, and absorption of ITCs are described according to the most recent findings. The focus of this review includes the hydrolysis reaction mechanisms, the elucidation of the main factors that play a role in the hydrolysis reaction, the influence of processing and cooking conditions, the effect of chewing, and the role of the gastric and upper intestinal phases, including the effect of the meal composition (e.g., the effect of other meal compounds present during digestion) on the potential formation of ITCs. The effect of the cultivation methods (e.g., soil composition, season, etc.) on GL formation in *Brassica*, the hydrolysis of GLs by the microbiota, and the health effects of BDPs are not included in this review, as they are the focus of other papers within this special issue.

## 2 Intrinsic Factors

### 2.1 Spatial Distribution of Glucosinolates and Myrosinase in Plant Tissues

The initial concentrations and chemical profiles of GLs differ among the *Brassica* plants,^23^ and within each plant, concentrations vary between the different organs.^24^ Varying GL concentrations were found in the inflorescences, siliques (fruits), leaves, and roots of *Arabidopsis thaliana*, with the highest levels observed in inflorescences and the lowest observed in the roots.^24^ Moreover, the concentrations of GLs are reported to decrease during developmental stages.^23^, ^24^

Studies have shown that GLs are stored in vacuoles of specific cells.^25^ In thale cress (*A. thaliana*), GLs were found in the so‐called ‘‘S‐cells’’ located between the phloem and the endodermis.^26^ The S‐cells are considered to be giant cells due to their large central vacuole surrounded by a thin layer of cytoplasm containing a few organelles and are adjacent to the so‐called myrosin cells, which contain MYR.^27^ Myrosin cells are present in seeds, roots, stems, leaves, and flowers and have been found in the main types of plant tissue such as vascular and epidermal tissues.^27^ Thus, in intact plant cells, MYR is physically separated from GLs. Plant tissue damage is the conditio sine qua non to start the enzymatic hydrolysis of GLs, allowing MYR and GLs to come into contact. For this reason, processing steps, cooking practices, and chewing all strongly affect the formation of BDPs and thus ITCs.

### 2.2 Mechanisms of GL Hydrolysis and ITC Formation

Plant tissue damage and the subsequent contact between MYR and GLs is not the only scenario capable of promoting ITC formation. As shown in Figure 1, other factors play a role in the formation of these compounds. A higher concentration of GLs does not always result in greater formation of ITCs upon hydrolysis. GL breakdown is a complex reaction guided by multiple factors affecting the MYR activity and/or the BDP profile.^28^ These factors fall into two distinct groups: factors affecting the hydrolysis of GLs into glucose and thiohydroximate‐*O*‐sulfonate by MYR (e.g., pH, temperature, metal ions, and ascorbic acid) and factors that affect subsequent thiohydroximate‐*O*‐sulfonate breakdown pathways that lead to different BDP profiles (e.g., pH and the presence of specific proteins such as the epithiospecifier proteins [ESP]).^28^

The first step of MYR (β‐thioglucosidase)‐catalyzed GL hydrolysis begins with the formation of an unstable aglycone of the thiohydroximate‐*O*‐sulfate. Studies have shown that l‐ascorbate acts as a cofactor for MYR by promoting the rate‐limiting step of the reaction.^29^ The aglycone either undergoes a Lossen rearrangement to form the corresponding ITC or forms the corresponding nitrile through a sulfur‐releasing degradation reaction.^30^, ^31^ Nitrile formation is favored at low pH values, as acidic protons hinder the Lossen rearrangement.^31^ The presence of ESP also favors conversion of the aglycone into nitriles or epithionitriles.^30^, ^32^ The activity of ESP was shown to be dependent on ferrous ions^33^ as well as on pH.^28^ For example, under acidic (pH 4) or alkaline (pH 8) conditions, the inactivation of ESP leads to higher ITC formation.^28^ If ESP is not present or is inactive, nitrile formation is favored at low pH, and under neutral or alkaline conditions, ITC formation increases.^31^ To understand, predict, and optimize the formation of ITCs, it is important to consider the effects of all factors and compounds involved.

## 3 Extrinsic Factors

### 3.1 Industrial and Domestic Processing

**Figure 2:**
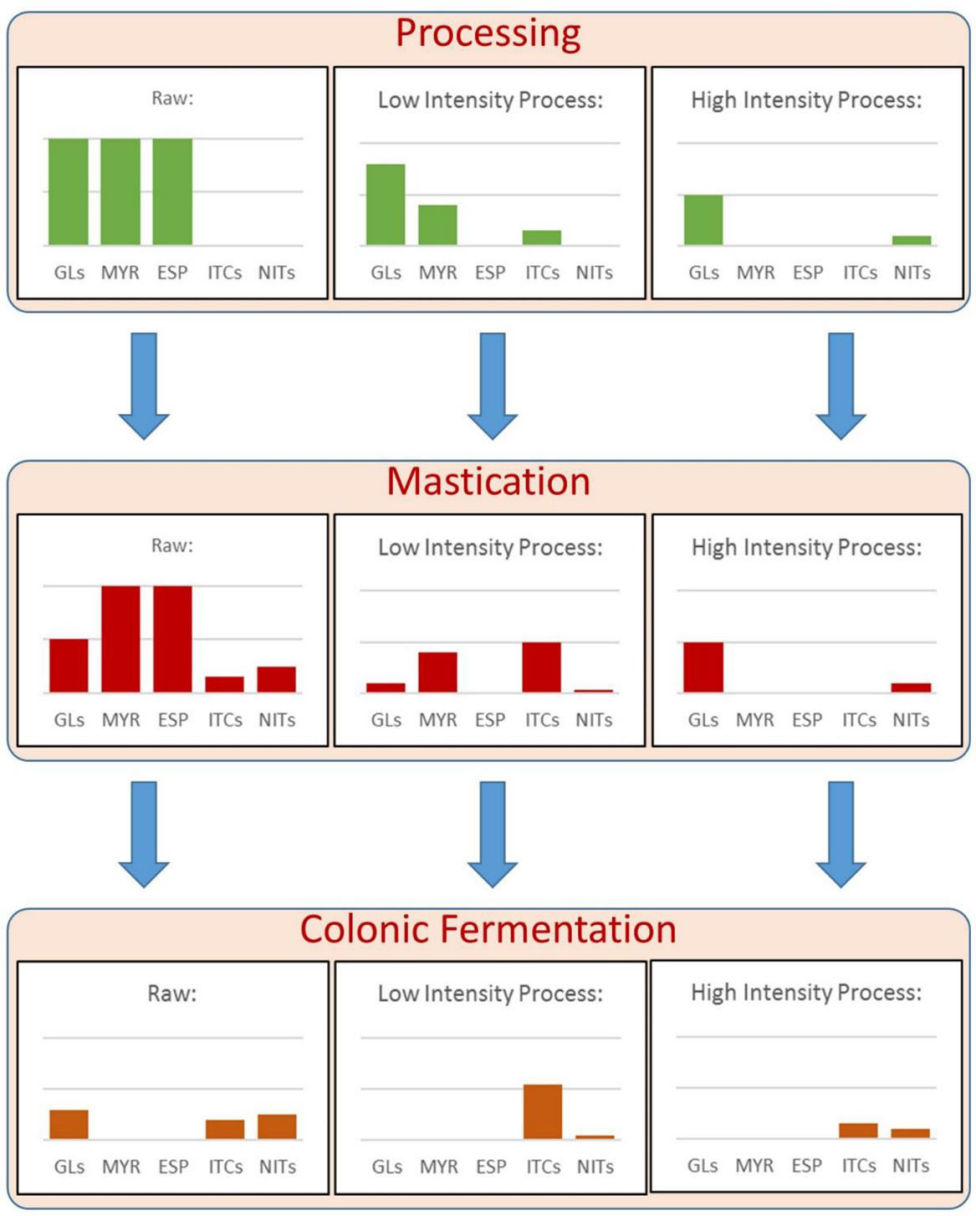
Schematic representation illustrating the expected effects of different processing intensities on the content and digestive formation of glucosinolates (GLs) and breakdown products (BDPs) by the mechanisms described in the text. The amount shown after colonic fermentation is the summation of the amount formed by the microbiota from intact GLs in the colon after chewing (in reality, BDPs present after chewing will be absorbed in the small intestine). The final amounts present after colonic fermentation represent the theoretical amounts that can be absorbed by the small intestine and colonic epithelial cells. In theory, mildly intensive processing that inactivates epithiospecifier proteins (ESP) but does not completely inactivate myrosinase (MYR) yields the highest amount of absorbed isothiocyanates (ITCs). Consuming raw vegetables primarily yields nitriles (NITs), which are not considered to be beneficial to health.

Industrial and domestic processing can dramatically affect ITC formation. Depending on the type of processing, the GL–MYR system is impacted by different mechanisms. Different processing conditions therefore have different effects on the terminal formation of ITCs (**Table** 1, **Figure** 2).^34^, ^35^ During processing, several mechanisms are present that influence the composition of GLs and BDPs in *Brassica* vegetables on the plates of consumers, including a) lysis of cells and cellular compartments and subsequent leaching of GLs and BDPs into the cooking water; b) enzyme‐catalyzed hydrolysis of GLs upon lysis and diffusion of GLs and MYR; and c) thermal degradation of GLs, inactivation of MYR, ESP, and thiocyanate‐forming protein (TFP), and loss of ascorbic acid and Fe^2+^.^34^ Cooking and industrial processing often make use of elevated temperatures that can lead to (partial) thermal degradation of GLs, BDPs, and ascorbic acid; the inactivation of MYR, ESP, and TFP; as well as cell lysis and subsequent leaching of compounds into the processing medium.^34^ Boiling is one process that greatly affects the aforementioned compounds. By immersing *Brassica* vegetables into boiling water for various lengths of time, depending on the desired consumer‐preferred final texture, the GL content can be substantially reduced. This reduction is caused by thermal degradation as well as by the leaching of components from the vegetable tissue into the boiling water.^34^, ^35^ The thermal degradation of vegetables during boiling can cause GL losses of 5–20%.^36^ Different GLs have different degradation rates. In red cabbage, the degradation rate constants of the indole GLs are reported to be significantly higher than the degradation rate constants of the aliphatic GLs at temperatures ≤110 °C (e.g., *k*_d,110 °C_ [×10^−3^ min^−1^] values are 11.5 ± 0.5 for glucoraphanin and 30.7 ± 0.3 for glucobrassicin).^23^ Different vegetable matrixes influence the thermal stability of GLs, suggesting that the plant matrix affects the rate of the degradation reaction.^25^, ^26^ In a study in which red cabbage, broccoli, brussels sprouts, pak choi, and Chinese cabbage were thermally treated, the degradation rate constants obtained for several glucosinolates vary between 4 and 20‐fold among the five vegetables.^37^ It is important to note that the thermal degradation of GLs in plant tissues leads primarily to the formation of nitriles, while in aqueous solutions ITCs are favored.^26^, ^27^

**Table 1:** Literature review on the effect of domestic and industrial processing applied to *Brassica* vegetables and their effect on the leaching (diffusion of components from the vegetable tissue to the boiling water), glucosinolates (GLs), and myrosinase (MYR). The effect on those factors is represented with symbols: (*) retained; (↓) reduced; (↓↓) highly reduced; (↓↑) depending on the treatment conditions different results can be obtained. The retention will depend on the conditions of each treatment; n.a., not affected; n.i., not investigated

Domestic or industrial treatment	Leaching	MYR	GLs	References
Air drying	n.a.	↓	*	63, 64
Freeze drying	n.a.	*	*	42, 57, 65
High pressure	n.a.	↓↑	↓↑	41, 66, 67, 68, 69, 70, 107, 108
Pulsed electric field	n.i.	n.i.	*	71
Boiling	↑↑	↓↓	↓↑	38, 39, 40, 47, 49, 51, 56, 109, 110, 111, 112
Microwaving	n.i.	↓↑	*	36, 39, 47, 54, 55, 111, 113
Steaming	n.a.	*	*	36, 38, 39, 40, 51, 53, 80, 110, 111, 113
Stir‐frying	n.a	n.i.	*↓	39, 48, 49, 56, 57, 114
Fermentation	n.i.	↓↓	↓	58, 60, 62

The extent of leaching depends on the water/vegetable ratio, the boiling time, the type and morphology of the vegetable tissues as well as the method used to chop the vegetables. This mechanism of loss has been confirmed by many studies in which the concentration of GLs was determined in both the vegetables and in the boiling water after cooking.^38^, ^39^, ^40^ In their review article, Nugrahedi et al. reported losses of 25–75% due to leaching.^34^ Apart from thermal degradation and leaching, the high temperature reached during boiling also inactivates MYR, preventing the formation of ITCs from the GLs still present in the vegetable.^34^ Researchers report that MYR isolated from broccoli is completely inactivated after 10 min of heating at 60 °C,^41^ whereas in the broccoli matrix the inactivation is completed after approximately 20 min at 60 °C.^42^ It is important to mention that even with complete inactivation of MYR, the remaining intact GLs are still beneficial for consumption, since intact GLs can also be hydrolyzed by the gut microbiome, although to a lesser extent, allowing ITCs to be formed during digestion.^43^, ^44^, ^45^

Steaming is a cooking method that makes use of saturated steam, allowing the vegetables to remain outside of the water bath. This method ensures that very little leaching of GLs into the cooking water occurs. Indeed, the results show that after steaming, the GLs are well‐retained,^34^, ^39^, ^46^, ^47^, ^48^ or their apparent concentration increased due to an increase of GL extractability after heating.^49^, ^50^ D'Antuono et al. reported a greater than twofold increase in total GL content in cauliflower after steaming,^50^ whereas Gliszczyńska‐Świglo et al. and Miglio et al. reported a 20–30% increase in the total GL content in broccoli.^49^, ^51^

Additionally, steaming may allow MYR activity to be partially retained.^46^ When steaming cabbage, the MYR activity was retained up until 2 min of heating time, while after 7 min, a loss of 90% was reported.^46^ Shorter steaming times can lead to a higher formation of ITCs over nitriles due to the inactivation of ESP that occurs at a lower temperature than the MYR inactivation as reported for broccoli.^52^, ^53^

Depending on the output powers and time of the treatment, microwave processing can be a suitable option to retain GLs. The application of microwaves generates heat that cooks the vegetable. Studies in which vegetables were microwaved without the addition of water report that GLs are retained after microwaving.^39^, ^54^ Microwave heating of cabbage in water at 750 W resulted in a 17.3% loss of total GL content after a 7 min treatment.^46^ A similar reduction (by 18%) was found by microwaving broccoli for 5 min at 1000 W,^55^ whereas under the same conditions a 74% reduction was reported in broccoli.^47^

Depending on the microwave power and length of time used, MYR can be partially or completely inactivated.^46^, ^54^ MYR activity in red cabbage was retained when treated for 24 min at 180 W and when treated for 8 min at 540 W, whereas MYR was inactivated when treated for 4.8 min at 900 W.^54^ The microwave treatment could also be a suitable process to enhance ITC formation by applying a low power (540 W) microwave treatment, since MYR is still active while ESP is inactivated.^52^

Stir‐frying is another commonly used cooking method in which the vegetables are fried with a small amount of preheated oil for a few minutes. Thermal degradation is the primary process observed during stir‐frying, since no water is added to the pan and no leaching into the cooking water can occur. Several studies report full retention of GLs after stir‐frying,^39^, ^56^, ^57^ but in other studies losses are reported.^48^ Stir‐frying “nero di Toscana” (*Brassica oleracea L*. var. *sabellica L*.) and “broccolo lavagnino” (*B. oleracea L*. var. *capitata L*.) for 20 min yielded a 65–75% reduction in total GLs, while applying the same methods to kale (*B. oleracea L*. var. *sabellica L*.) afforded an approximately 30% reduction.^48^ Although the high temperatures reached during stir‐frying may dramatically reduce MYR activity,^42^ the retained GLs can still be converted into ITCs by the enzymatic activity of the microbiota.^43^, ^44^, ^45^

Treatments which do not involve the use of high temperature can also reduce the GL–MYR system. Fermentation is a traditional processing method used to produce fermented vegetables such as sauerkraut. During fermentation, lactic acid bacteria grow in salted vegetables under optimized conditions (usually anaerobically at room temperature). After 7 days of fermentation, complete GL degradation was reported in white cabbage^58^ and a 13‐fold reduction was reported in nozawana leaves (*Brassica campestris L*. var. *rapifera*).^59^ These reductions can be explained by the long incubation time in water that may have favored MYR‐catalyzed GL hydrolysis after cell lysis. In pretreated MYR‐inactivated vegetables, 13% of the total GLs were retained after 7 days of fermentation.^60^, ^61^ BDPs can also be lost during fermentation; indole‐3‐carbinol (BDP of glucobrassicin) levels in high‐pressure treated sauerkraut were reduced by 7% after 3 months of refrigerated storage.^62^

Other processing methods that are used by the food industry can also affect the GL–MYR system. Air drying may retain GLs depending on the treatment conditions (water content, temperature, and time).^63^, ^64^, ^65^ Various air drying temperatures (50–100 °C) led to varying extents of reduction (17–45%) of total indole‐containing GLs in broccoli,^64^ while the more heat labile MYR was completely inactivated.^42^, ^57^, ^63^, ^64^, ^65^ However, by optimizing the conditions, all of the glucoraphanin and 55% of the endogenous MYR could be retained upon air drying of broccoli.^63^

High‐pressure processing is a method that has also been extensively investigated for the GL–MYR system. This process uses the combined effects of pressure and temperature to inactivate the enzymes and reduce the microbial count of produce and is meant to be a milder alternative to pasteurization or sterilization. The advantage is that it affords the opportunity to selectively inactivate certain enzymes while retaining other enzymes such as MYR, and ruptures cell membranes to allow the GLs to come into contact with MYR.^66^, ^67^, ^68^, ^69^ High‐pressure treated broccoli (600 MPa for 3 min at 30 °C) contained a 6‐fold higher ITC concentration compared to the untreated samples.^70^ Another new technology is pulse electric field processing. This technology applies electric pulses to breakdown cell membranes and is also meant as a milder alternative to pasteurization. This technology can potentially be applied to cell membrane degradation, enhancing contact between MYR and GLs to form ITCs. However, the treatment requires sample immersion in water and a temperature elevation that may lead to the leaching of GLs and ITC. Up until now, only one study has investigated the effect of a pulse electric field on the GL–MYR system, and only the GL concentration was monitored.^71^ Following the treatments (1–4 kV cm^−1^, 50–1000 μs), the GL concentrations increased remarkably (110.6–212.5%), most likely due to an increase in cell permeability that increased the extraction yield.^71^

Freezing is performed at around −20 °C and is used to increase the shelf life of vegetables. Prior to freezing vegetables, a blanching treatment is usually applied to inactivate the enzymes that would otherwise reduce the quality of the frozen product. After 3 months of storage at −20 °C, the MYR activity and GL content of blanch‐frozen broccoli were not affected, except for neoglucobrassicin.^56^ In blanch‐frozen cauliflower, GL retention (both aliphatic and indolic GLs) was observed even after 12 months of storage. However, freezing the vegetables (−85 °C for 7 days) without prior blanching led to a 33% loss of total GL content.^56^ According to the authors, the reduction of GL hydrolysis could be ascribed to the MYR‐catalyzed hydrolysis during thawing of the frozen vegetables.^56^ During freezing, water crystallizes in extracellular and intracellular spaces, causing cell membrane damage and leading to the MYR‐catalyzed hydrolysis of GLs during thawing.^39^, ^72^

Cold storage at 4–8 °C for 7 days affected the GL concentration in broccoli, brussels sprouts, cauliflower, and green cabbage. Reductions of 27%, 20%, 11%, and 14%, respectively, were reported.^39^

Cooking vegetables in the presence of other food compounds is an extrinsic factor that may affect the GL–MYR system, particularly the thermal degradation of GLs, and has received less attention than the previously discussed topics. This factor was investigated in a study using freeze‐dried broccoli powder in which MYR was previously inactivated. The broccoli powder was mixed with either potato starch, corn starch, lentil proteins, or freeze‐dried onion to create binary systems.^73^ The observed thermal degradation in all of the mixed samples was lower than the non‐mixed control. Most strikingly, the system containing onions exhibited GL retention levels 2‐fold higher than those observed for the sample containing only broccoli.

*Brassica* vegetables can be used as food ingredients to enrich products that traditionally do not contain such ingredients. From a technological point of view, there are products, such as pasta (e.g., spaghetti) and bread that are easily enriched with ingredients such as dried *Brassica* powder. For instance, consumption of 100 g (DW) of pasta enriched with broccoli (16% broccoli addition on a dry weight basis) affords an intake similar to a portion of fresh broccoli (approximately 200 g).^74^ However, when making the enriched pasta, the pasta drying and cooking process can affect the GL–MYR system, reducing the amount of GLs present in the final meals of consumers.^74^

### 3.2 In the Human Body

#### 3.2.1 Pharmacokinetics

Bioaccessibility and Bioavailability: The final ITC or GL intake depends on the vegetable preparation/processing methods (e.g., cell lysis, GL concentration, MYR activity, ESP activity) as described in the previous section. In general, the bioaccessibility of a compound is the fraction of that compound that reaches the site of absorption and that can potentially be absorbed by the intestinal epithelial cells or through tight junctions. The bioavailability of a compound is the fraction of that compound that is actually absorbed and circulates systemically to organs and tissues and is used for physiological functions. However, for the GL–MYR system, the definitions of bioaccessibility and bioavailability become more complex since the GLs are most often converted into their BDPs for absorption, rather than being absorbed themselves.

The fraction of GLs that is released from the vegetable matrix can be defined as bioaccessible/available for hydrolysis by the endogenous MYR in plants or by the MYR‐like activity of the human microbiota (Figure 2).^43^, ^44^, ^45^

The bioaccessible ITC fraction represents the fraction that reaches the absorption site (release of the ITCs from the food matrix), and the bioavailable fraction represents the ITC fraction that is absorbed and achieves systemic circulation. To calculate these fractions, the amount of ingested ITCs and those formed from GLs inside the human body (during chewing or in the lower gut) should be taken into account.

**Figure 3:**
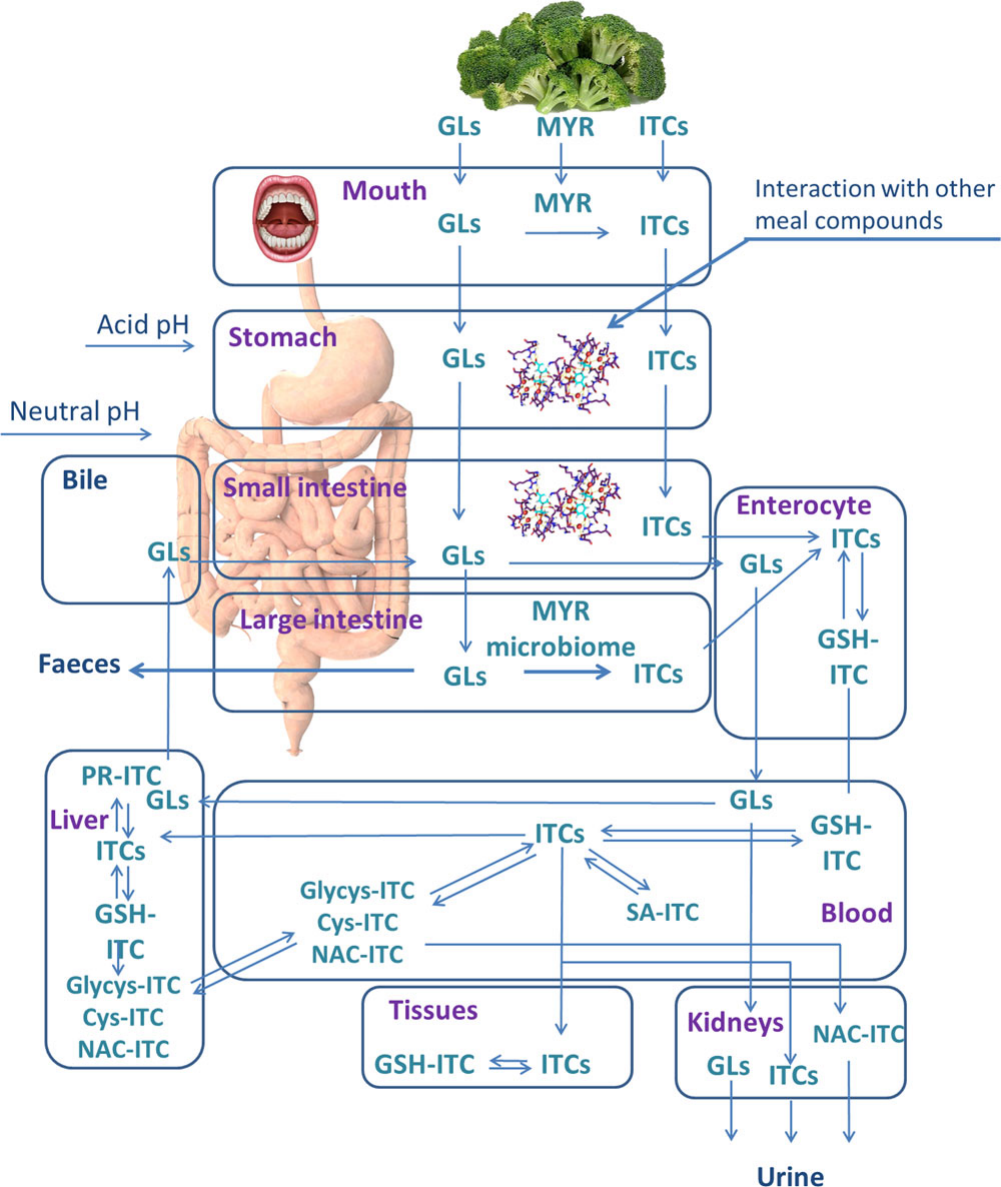
Overview of the metabolic fates of glucosinolates (GLs) and isothiocyanates (ITCs). MYR, myrosinase; GSH‐ITC, glutathione conjugate of ITC; PR‐ITC, intracellular proteins conjugates of ITC; SA‐ITC, serum albumin conjugate of ITC; Nac‐ITC, *N*‐acetylcysteine conjugate of ITC; glycys‐ITC, glycine‐cysteine conjugate of ITC; cys‐ITC, cysteine conjugate of ITC; Pr‐ITC, intracellular proteins conjugates.

*Absorption*: The actual beneficial effects of a compound depend on the amount present in the food, as well as on the bioaccessibility and the bioavailability of that compound. Compounds that are bioaccessible become bioavailable when the compounds are absorbed. Studies show that a small portion of the intact GLs can be absorbed and excreted intact, although the absorption mechanism is unclear (passive diffusion or facilitated transport) (**Figure** 3).^75^, ^76^ The portion of GLs that arrive in the lower gut is bioaccessible for MYR‐like activity of the human microbiota, although the formation of ITCs is reported to be lower than the conversion that occurs under plant conditions (Figure 3).^56^, ^57^, ^58^

Both of the fractions of ITCs that are already present in food or are formed during chewing and those that are formed in the lower gut are absorbed by the epithelial cells of the small intestine or colon (Figure 3). After conjugation with the thiol group of the glutathione cysteine residue, the ITC conjugates are transported out of the cells into the extracellular space through active transport by multidrug resistance‐associated protein 1, 2 and P‐glycoprotein.^77^, ^78^ In the blood, ITC‐glutathione conjugates dissociate due to the low glutathione plasma concentration and the free ITCs can conjugate with serum albumin, which is the most abundant source of available free thiol groups. Free ITCs in equilibrium with the ITC conjugates are absorbed by peripheral organs and accumulate in cells by reacting with thiol groups of glutathione and proteins. Free ITCs and ITC conjugates are ultimately excreted in the urine.^79^, ^80^, ^81^ Therefore, to study the bioavailability of ITCs, concentrations of free ITCs and the ITC metabolites can be obtained through plasma and urine sample analysis.^76^, ^82^, ^83^ More detailed studies are needed to understand the pharmacokinetics and bioactivity of ITCs.

#### 3.2.2 Oral Phase—Mastication

Plant MYR‐activated GL hydrolysis begins at the moment of consumption with the onset of chewing. During the act of chewing, the vegetable tissue is crushed and ground, and the released compounds are mixed with saliva within the food bolus. ITC formation during chewing is dependent on the chewing intensity, the concentration of GLs, MYR, and all the factors that influence the reaction (**Table** 2, Figures 1 and 2). To study the formation of sulforaphane and sulforaphane nitriles (BDPs of the GL glucoraphanin) during chewing, broccoli samples were steamed for 0.5, 1, 2, and 3 min to obtain broccoli with different MYR and ESP activities. The samples were chewed for various lengths of time by human volunteers for 11–40 s at a standardized rate of 1 chew s^−1^.^84^ Upon completion of chewing, concentrations of glucoraphanin, sulforaphane, and sulforaphane nitrile concentrations were measured. Length of chewing time did not significantly affect formation of sulforaphane and sulforaphane nitriles. However, the samples steamed for 2 min led to significantly greater formation of sulforaphane (2‐fold higher) after chewing then the raw broccoli control and the broccoli steamed for 3 min.^84^ These results confirm the hypothesis that ESP inactivation prior to MYR inactivation (after low intensity steaming) enhances the formation of ITCs.^85^ However, additional studies should be performed to investigate how individual chewing practices may affect ITC formation. The individual differences in chewing methods may help to explain the high variability in ITC metabolite excretion reported during many in vivo studies in which volunteers consumed broccoli.^45^, ^80^, ^86^, ^87^, ^88^

**Table 2:** Literature review on the effect of digestion (in vitro or in vivo) and co‐digestion (digestion along with other food compounds) on the bioaccessibility (BioAc) or bioavailability (BioAv) of glucosinolates (GLs) and isothiocyanates (ITCs) in *Brassica* vegetables differently treated or as isolated compounds. In all the studies, the bioavailability was calculated by analyzing the excretion of the mercapturic acids of the corresponding ITCs in urine. The changing of bioaccessibility or bioavailability is represented with symbols: (–) no change; (↓) decreased; (↑) increased; n.i., not investigated

Compound/vegetable	Type of study	Treatment	BioAc	BioAv	References
GLs (not identified)/roots of Chinese red radish	in vitro	Acid pH	↓	n.i.	90
Glucobrassicin/synthetized	in vitro	Neutral pH Acid pH	↓ ↓	n.i.	115
Progoitrin, gluconapoleiferin, gluconapin, 4‐Hydroxyglucobrassicin, glucobrassicanapin/rapeseed meal	in vitro	Acid pH Neutral pH	↓ ↓	n.i.	92
Progoitrin, glucoraphanin glucoalyssin, gluconapin, 4‐hydroxyglucobrassicin, glucobrassicin, 4‐methoxyglucobrassicin/broccoli	in vitro	Acid pH Neutral pH	↓ ↓	n.i.	91
Glucoraphanin, allyl ITC/mustard and broccoli (differently cooked)	in vivo	Consumption with and without beef	n.i.	↑ for allyl ITC	100
Glucoraphanin, glucoiberin/broccoli sprouts	in vivo	Consumption with proteins or dietary fibers or lipids gels	–		96
Sulforaphane/broccoli (raw or differently steamed)	in vitro	Acid pH Neutral pH Co‐digestion with oil or protein	↑ ↑ No clear trend	n.i.	53
Sulforaphane/broccoli (raw or differently steamed)	in vitro	After the intestinal phase	↓	n.i.	93
Glucoraphanin/broccoli (raw or differently steamed)	in vitro	Acid pH Neutral pH Co‐digestion with oil or protein	– – –	n.i.	53
Glucoraphanin/broccoli (raw or differently steamed)	in vivo	Mastication	↓		84
Sulforaphane/broccoli (raw or differently steamed)	in vivo	Mastication	↑	n.i.	84
Sulforaphane, iberin/broccoli sprouts	in vivo	Consumption with proteins or dietary fibers or lipids gels	n.i.	↓	96

Chewing may particularly affect the bioaccessibility of compounds present in intact consumed food (not ground or smashed) in which the compounds are encapsulated in the cells. The efficiency of chewing will be affected by the applied cooking treatment that will affect the resulting vegetable texture. Thus, the interaction between chewing practices and the vegetable texture is important to consider. In raw crunchy fruits and vegetables, cell adhesion is stronger and fractures of the plant tissue occur mainly through cell walls. Hence, during chewing, the reported number of fractured cells is relatively high.^89^ In cooked fruits and vegetables, the cell adhesion is weaker due to pectin solubilization, and tissue fractures occur mostly along cell walls. This occurrence leads to the production of intact cell clusters during chewing which encapsulate the compounds.^89^ Alternatively, cell wall and membrane damage may occur during cooking, allowing for diffusion of GLs and MYR. Therefore, implementation of the in vitro digestion protocol with a chewing step resembling authentic human chewing is important when investigating the bioaccessibility of compounds in vitro, especially when studying the conversion of GLs to ITCs. These studies often exert more effort in mimicking the gastric and intestinal phases and simply replace the chewing step with mincing, grinding, or blending. These techniques produce much smaller particle sizes than those formed upon in vivo chewing, and can lead to overestimation of the bioaccessibility of compounds that are released and formed during chewing.

#### 3.2.3 Gastric and Intestinal Phase

Studies have reported the stability of GLs during in vitro digestion and the amount of intact GLs that can pass through the gastric and intestinal phases (Table 2). The stability of the total GLs (extracted from roots of the Chinese red radish) was investigated at various pH levels. It was shown that at pH values ranging from 3.6 to 9.1, 88–97% of the GL initial content was retained, while at pH less than 3.6 the retention was reduced, and at pH 1.5 only 60% of GLs were retained.^90^ Similar reduction of the total GLs (69%) was also reported in broccoli under gastric conditions of in vitro digestion (pH 2) and an additional 12% of GL reduction was reported under intestinal conditions (pH 7.8).^91^ However, different GLs exhibited different stabilities during digestion. During gastric in vitro digestion of rapeseed meals, the concentrations of progoitrin and gluconapoleiferin declined by 18% and 23%, respectively, whereas gluconapin and glucobrassicin declined by only 3% and 8%, respectively, and 4‐hydroxyglucobrassicin was no longer detectable.^92^ Under small intestine–like conditions, the loss of GLs were reported to be 23%, 7%, 17%, and 28% for progoitrin, gluconapoleiferin, gluconapin, and glucobrassicanapin, respectively.^92^ It is important to mention that Maskell et al. and Vallejo et al. studied samples in which MYR was still active, and this may have influenced the reduction of GLs, although at the acidic pH of the gastric phase, MYR should have been denatured.^92^ During in vitro digestion of broccoli samples that were steamed for different time intervals, glucoraphanin concentration did not change significantly regardless of the steaming treatment.^53^ The authors concluded that in some samples, although MYR activity was detected after steaming, no further hydrolysis occurred during digestion due to the acidic pH of the gastric phase.^53^

Little is known about the stability of the ITCs (and other GL BDPs) during digestion (Table 2). During in vitro digestion, up to 50% of sulforaphane (ITC from glucoraphanin) was released from the broccoli matrix and could be collected in the bioaccessible dialyzed fraction, while a small amount remained in the non‐dialyzed fraction and a large amount of sulforaphane was not recovered in either fraction at the end of digestion.^93^ Other results show that the content of sulforaphane and sulforaphane nitrile in the digestive fluids (both in the gastric and intestinal phases) increased during the digestion of raw or 1 min‐steamed broccoli, although no reduction of glucoraphanin concentration was seen in the same digested samples.^53^ The authors explained the increase of BDPs during digestion as the continuous release of BDP compounds from the broccoli matrix rather than additional formation.^53^ At the acidic pH of the gastric phase, indole‐3‐carbinol, the main BDP of glucobrassicin, can form dimers and condensation products such as diindolylmethane or indolo[3,2‐*b*]carbazole.^94^

#### 3.2.4 Meal Composition

Another factor that may affect the bioaccessibility and bioavailability of GLs and ITCs is meal composition. Dietary fiber is known to form complexes with other dietary components, for example, polyphenols and phenolic acids,^95^ and the same may occur for GLs or ITCs.

In an exploratory in vivo study of the effect of food compounds on ITC bioavailability, the incorporation of heated broccoli sprouts into gels of proteins, dietary fibers, or lipids led to higher glucoraphanin and glucoiberin bioaccessibility and bioavailability in vivo than consuming heated (MYR‐inactivated) broccoli sprouts alone, although these results were unconfirmed by statistical tests.^96^ The hydrolysis of glucoraphanin and glucoiberin by the MYR‐like activity of the gut microbiota was confirmed by a typical delayed excretion peak of the ITC metabolites that occurred 3–6 h later than the excretion peak resulting from ingestion of already formed ITCs.^44^, ^86^, ^87^, ^88^, ^97^, ^98^ In the same study, the opposite trend was observed when volunteers consumed gels in which sulforaphane and iberin were already preformed by active MYR. The incorporation of broccoli sprouts in gels of proteins, dietary fibers, or lipids led to lower bioavailability of sulforaphane and iberin compared to the control broccoli sprout (the same treated sprouts not incorporated in the gels).^96^ However, for sulforaphane, this trend was not confirmed by statistical tests, while for iberin a higher bioavailability of the control was confirmed only for the protein gel. The study was an exploratory study containing five participants; a new study with a greater number of participants should be performed to confirm these results.

The in vitro co‐digestion of raw broccoli or broccoli steamed for different amounts of time with protein or lipids led to significant differences in the bioaccessibility of sulforaphane, but not for glucoraphanin and sulforaphane nitrile.^53^ Sulforaphane concentrations in raw and 1‐min steamed broccoli digested with added protein were 25–26% higher compared to the same samples that were digested with added lipids or with no addition to the broccoli.^53^

Animal studies show that the bioavailability of allyl‐ITC (the ITC of sinigrin) is higher when it is ingested with milk or corn‐oil (1.4–1.8‐fold).^99^ In a human study, the bioavailability of allyl‐ITC was found to be 1.3‐fold higher when broccoli was co‐ingested with meat.^100^ This effect was explained by the presence of fat in meat that may have enhanced the absorption of ITCs due to the incorporation of ITCs into mixed micelles.^100^ The ITCs are electrophilic compounds that can react with compounds containing thiol, hydroxyl, and amino groups such as amino acids, peptides, and proteins. In vitro studies show that ITCs can potentially react with amino acids, peptides, and proteins to form a vast range of thiocarbamates, dithiocarbamates, and thiourea derivatives, and this reactivity may reduce the ITC bioavailability in protein‐rich foods.^101^, ^102^, ^103^, ^104^

## 4 Future Perspectives

Numerous intrinsic and extrinsic factors can affect the final intake, formation, bioaccessibility, and bioavailability of ITCs. Great efforts have been made to breed *Brassica* vegetables containing a higher GL content in order to understand the effects of industrial and domestic processing on the GL–MYR system, to optimize these processes, to estimate the bioaccessibility and bioavailability of ITCs, and to investigate the beneficial health effects of ITCs in vitro and in vivo. However, there are still many mechanisms and factors that need to be investigated in greater detail. Factors that affect the bioaccessibility/bioavailability of GLs and ITCs such as meal composition are not yet clear and more studies should be performed to elucidate how the final ITC absorption can be enhanced during consumption of a meal, especially considering that *Brassica* vegetables are primarily consumed as part of a meal. Concerning the biological activity of the ITCs, many researchers have investigated their beneficial health effects in vitro; however, more in vivo studies should be performed to confirm the outcome obtained in vitro and to define a recommended intake of ITCs per day or per week.

## Conflict of Interest

The authors declare no conflict of interest.
